# Multi‐Omics analysis identifies that different genetic perturbations affect various molecular mechanisms underlying Alzheimer's Disease (AD) in an age‐dependent manner

**DOI:** 10.1002/alz70855_096942

**Published:** 2025-12-23

**Authors:** Ravi S Pandey, Kevin P Kotredes, Dylan Garceau, Catrina Spruce, Anantharaman Shantaraman, Duc M Duong, Carolyn Paisie, Nicholas T Seyfried, Claudia Rangel‐Barajas, Michael Sasner, Adrian L Oblak, Gareth R Howell, Bruce T. Lamb, Gregory W Carter, MODEL‐AD CONSORTIUM

**Affiliations:** ^1^ The Jackson Laboratory, Bar Harbor, ME, USA; ^2^ Emory University, Atlanta, GA, USA; ^3^ Emory University School of Medicine, Atlanta, GA, USA; ^4^ The Jackson Laboratory for Genomic Medicine, Farmington, CT, USA; ^5^ Stark Neuroscience Research Institute, Indianapolis, IN, USA; ^6^ Stark Neurosciences Research Institute, Indiana University School of Medicine, Indianapolis, IN, USA; ^7^ Indiana University School of Medicine, Indianapolis, IN, USA

## Abstract

**Background:**

Alzheimer's disease (AD) is a complex, multifactorial pathology characterized by high heterogeneity in biological alterations. New genetic and genomic resources are identifying multiple genetic risk factors for late‐onset Alzheimer's disease (LOAD). However, our understanding of the cellular and molecular mechanisms linking disease risk variants to various phenotypes remains limited. Therefore, it is essential to integrate information from multiple data modalities to thoroughly explore endophenotype networks and biological interactions related to the disease, thereby accelerating our understanding of heterogeneity in Alzheimer's disease.

**Method:**

We obtained transcriptomics and proteomics data from whole hemibrain samples of mouse models harboring the genetic risk variants *Abca7^A1527G^, Mthfr^677C>T^, and Plcg2* ^M28L^. These mouse model already carrying humanized amyloid‐beta*, APOE4, and Trem2* ^R47H^ alleles, all knocked into a C57BL/6J background. *We* included mouse models of multiple ages for both sexes. Using state‐of‐the‐art bioinformatics tools, we conducted multi‐omics analysis to identify molecular alterations in these mouse models. Furthermore, we systematically aligned the multimodal mouse data with relevant human study cohorts to determine the AD relevance of risk genes.

**Result:**

The effects of these genetic variants recapitulated a variety of human gene and protein expression patterns observed in the LOAD study cohort. The Abca7 variant exhibited extracellular matrix, neuroimmune, and oligodendrocyte‐related gene signatures at an early age, correlating with postmortem LOAD cases compared to controls. By 18 months of age, the Mthfr variant exhibited vasculature, myelination, and synapse‐related gene and protein signatures, also correlating with postmortem LOAD cases relative to controls. The Plcg2 variant exhibited neuroimmune, endolysosome, and synapse‐related gene signatures and altered cell‐ECM interaction processes at the protein level, correlating with postmortem LOAD cases compared to controls.

**Conclusion:**

We have characterized in vivo signatures of three genetic candidates for late‐onset Alzheimer's disease (LOAD), identifying alterations in specific LOAD‐related pathways for each variant. Our study highlights that assembling multi‐omics measurements reveals interrelated pathway alterations in Alzheimer's Disease (AD) and enables the identification of biomarker combinations that may inform clinical practice. Our approach provides a platform for further exploration into the causes and progression of AD by assessing animal models at different ages and/or with different combinations of LOAD risk variants.

